# Induction of cystine/glutamate transporter in bacterial lipopolysaccharide induced endotoxemia in mice

**DOI:** 10.1186/1476-9255-4-20

**Published:** 2007-09-26

**Authors:** Kumiko Taguchi, Michiko Tamba, Shiro Bannai, Hideyo Sato

**Affiliations:** 1 Department of Biochemistry, Institute of Basic Medical Sciences, University of Tsukuba, Tsukuba, Ibaraki, Japan; 2 Department of Bioresource Engineering, Faculty of Agriculture, Yamagata University, Tsuruoka, Yamagata 997-8555, Japan

## Abstract

**Background:**

Cystine/glutamate transporter, system xc-, contributes to the maintenance of intracellular glutathione levels and the redox balance in the extracellular space. The main component of the transporter, xCT, is known to be strongly induced by various stimuli like oxidative stress in mammalian cultured cells. We examined the expression of xCT mRNA *in vivo *in the experimental endotoxemia.

**Methods:**

Northern blot analysis and *in situ *hybridization were used to investigate the expression of xCT mRNA in the tissues of the mice exposed to bacterial lipopolysaccharide (LPS).

**Results:**

Northern blot analysis revealed that xCT mRNA was constitutively expressed in the brain, thymus, and spleen, and that the expression of xCT mRNA was strongly up-regulated in thymus and spleen by the administration of a sublethal dose of LPS. In addition to brain, thymus, and spleen, xCT mRNA was detected also in the bronchiolar epithelium of the lung by the administration of the lethal dose of LPS.

**Conclusion:**

xCT is induced in some specific tissues by the administration of LPS. The results suggest that cystine/glutamate transporter plays an important role under the inflammatory conditions.

## Background

Sepsis is a severe disorder associated with high lethality in men even under appropriate treatment with antibiotics and complete eradication of bacteria. Lipopolysaccharide (LPS)-induced endotoxemia is a well-established model for infection with Gram-negative bacteria. LPS induces symptoms such as fever, hypotension, disseminated intravascular coagulation, and multiple organ system failure, and thus mimics sepsis caused by bacteria. Recent studies have indicated that distinct Toll-like receptors (TLR) are the key molecules recognizing either Gram-negative or Gram-positive bacteria [1]. LPS binds to TLR4 on leukocytes, triggering a cascade of downstream events including synthesis and release of cytokines like tumor necrosis facter-a (TNF-a) and interleukins [2].

Cystine/glutamate transporter, designated system x_c_^-^, is an exchange agency for anionic amino acids with high specificity for the anionic form of cystine and glutamate [3]. This transporter is known to contribute to the maintenance of intracellular GSH levels in many types of mammalian cells in culture [4]. It has also been suggested that this transporter contributes to the maintenance of the redox balance out of the cell [5]. Cystine/glutamate transporter consists of two protein components, xCT and the heavy chain of 4F2 antigen (4F2hc), whereas the transport activity appears to be mediated by xCT [6]. The activity of cystine/glutamate transporter is induced by various stimuli, including electrophilic agents like diethyl maleate, oxygen, hydrogen peroxide, amino acid deprivation, TNF-a, and LPS [7-9]. We have demonstrated that the induction of xCT mRNA by diethyl maleate is mediated through the electrophile response element (EpRE) (also called antioxidant response element). The EpRE is located in the 5' flanking region of xCT gene and activation of xCT gene transcription involves the transcription factor nuclear factor-erythroid 2-related factor 2 (Nrf2), which binds to the EpRE element [10].

xCT mRNA and the activity of cystine/glutamate transporter is dramatically induced in mouse peritoneal macrophages by LPS even at very low concentrations, similar to that as observed in the plasma of patients with sepsis [9]. Unlike diethyl maleate, LPS induction of xCT expression involves a different mechanism and is not mediated by the EpRE [10]. We have shown that xCT mRNA is constitutively expressed in specific regions of the brain [11], but not in liver, heart, kidney, and lung [6]. Recent studies indicated that xCT plays a pivotal role in the brain [12-15] and immune system [16]. It has, however, remained enigmatic whether LPS induces xCT expression also *in vivo*. Therefore, we have investigated the effect of LPS on the induction of xCT mRNA in mice under the pathological conditions like sepsis. Our results show that xCT mRNA is strongly up-regulated in thymus, spleen and lung in response to LPS, suggesting that system x_c_^- ^plays an important role under inflammatory conditions.

## Methods

### Materials and Animal protocols

Total RNA was isolated from C57BL/6J male mice aged at 8–12 weeks. LPS from S. typhosa (DIFCO Laboratories) was resuspended in sterile saline. Endotoxemia was induced by intraperitoneal injection of different doses of LPS ranging from 0.05 to 160 mg/kg. The RNA probes for mouse xCT, mouse 4F2hc, and mouse β-actin were digoxigenin (DIG)-labeled by transcription from the linearized plasmids using RNA-labeling mix (Roche) and T3/T7 RNA polymerase (Stratagene).

The experimental procedures involving animals were approved by the University of Tsukuba Animal Care and Use Committee and were done in accordance with its guidelines.

### Northern blot analysis

Total RNA was extracted using Isogen (Nippon Gene, Japan). The RNA was electrophoresed on a 1% agarose gel in the presence of 2.2 M formaldehyde, transferred onto positively charged nylon membrane (Roche), and hybridized with the DIG-labeled RNA probes in DIG Easy Hyb (Roche) for 16 h at 68°C. The membranes were washed twice for 5 min at room temperature with 1 × SSC, 0.1% SDS and then washed twice for 15 min at 68°C with 0.1 × SSC, 0.1% SDS. The hybridized bands were visualized using CDP-Star (Roche).

### *In situ *hybridization

The expression of xCT mRNA in the tissues was detected by *in situ *hybridization as described previously [11]. Briefly, mice were anesthetized with sodium pentobarbital (20 mg/kg, i.p.), perfused, and fixed with 4% paraformaldehyde in phosphate-buffered saline (PBS), pH 7.4. The tissues were excised and post-fixed in the same fixative overnight. Then, the tissues were incubated in 30% sucrose in PBS overnight and embedded with optimal cutting temperature compound (Sakura Finetechnical Co., Ltd., Tokyo, Japan). Sections (10 μm) were cut in a cryostat. The slides were placed in PBS containing 0.1% Tween 20 (PBT) at room temperature twice for 5 min and then incubated in PBT containing 1 mg/ml proteinase K at 37°C for 5 min. The slides were rinsed in PBT three times, fixed with 4% paraformaldehyde, rinsed in PBT three times, and incubated with hybridization buffer (50% formamide, 5 × SSC, pH 4.5, 1% SDS, 50 μg/ml heparin, and 50 μg/ml yeast RNA). After the addition of the DIG-labeled probe (1000 ng/ml), slides were hybridized at 65°C overnight. Slides were rinsed in 50% formamide, 5 × SSC, pH4.5, 1% SDS at 65°C for 30 min, in 50% formamide, 2 × SSC at 65°C for three times for 30 min each, and in 25 mM Tris-HCl, pH 7.5, 0.8% NaCl, 0.02% KCl, 0.1% Tween 20 (TBST) at room temperature three times for 5 min each. Slides were submerged in the blocking buffer [0.5% blocking reagent (Roche) in TBST] at room temperature for 1 hr and then incubated in sheep anti-DIG antibody conjugated to alkaline phosphatase in the blocking buffer at 4°C overnight. Then, the slides were rinsed and developed in the dark in BM purple alkaline phosphatase substrate solution (Roche) containing 2 mM levamisole for 2 days.

## Results

**Figure 1:**
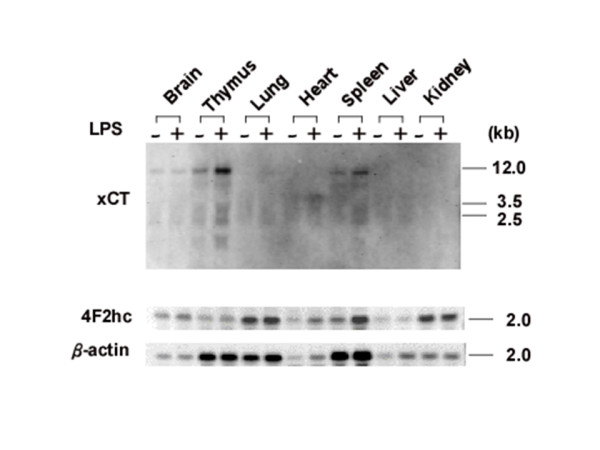
**Tissue distribution of xCT and 4F2hc mRNA in mice injected with or without LPS**. Mice were intraperitoneally injected with saline (-) or LPS 0.5 mg/kg LPS (+), and tissues were isolated 8 h after administration. Total RNA was extracted and Northern blot analysis was performed using DIG-labeled RNA probes.

**Figure 2:**
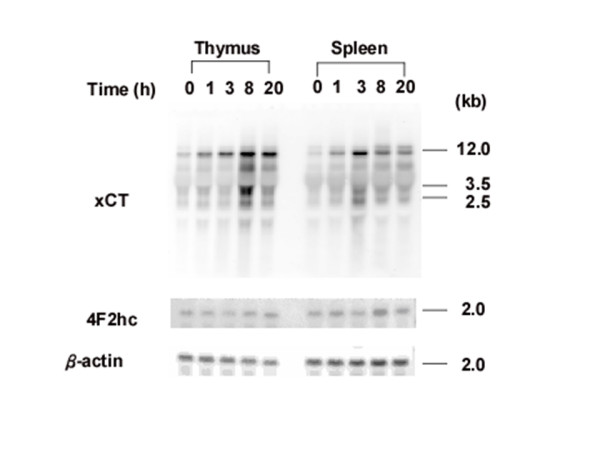
**Time course of expression of xCT and 4F2hc mRNA in thymus and spleen of the mice injected with LPS**. Mice were intraperitoneally injected with 0.5 mg/kg LPS, and thymus and spleen were isolated at the indicated time points. Total RNA was extracted and Northern blot analysis was performed using DIG-labeled RNA probes.

**Figure 3:**
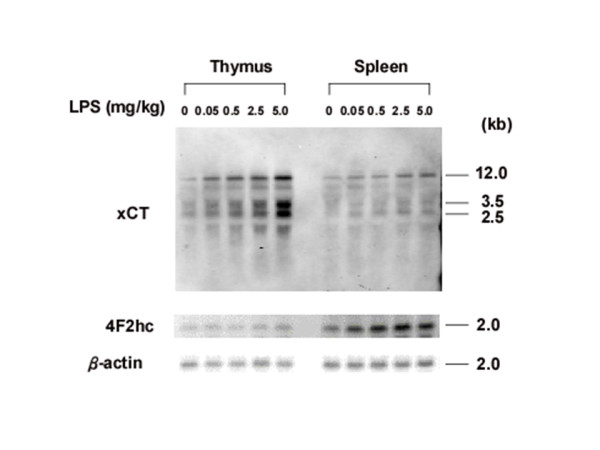
**Dose-dependent expression of xCT and 4F2hc mRNA in thymus and spleen of the mice injected with LPS**. Mice were intraperitoneally injected with LPS at the dose indicated, and thymus and spleen were isolated after 8 h. Total RNA was extracted and Northern blot analysis was performed using DIG-labeled RNA probes.

To examine basal and inducible expression of the mouse xCT gene in vivo, C57BL/6J mice were injected intraperitoneally either with saline or with saline containing LPS (0.5 mg/kg body weight). Total RNA was extracted from mice tissues and analyzed by Northern blotting with mouse xCT and 4F2hc DIG-labeled RNA probes. Results from a representative animal are shown in Fig. 1. In the control mouse (saline injection), the xCT mRNA was detected in brain, thymus, and spleen, but not in lung, heart, liver, and kidney. LPS administration significantly increased the expression of xCT mRNA in thymus and spleen. The constitutive expression of xCT mRNA in the brain was faintly increased by LPS. To investigate the kinetics of xCT mRNA up-regulation in thymus and spleen, mice were injected intraperitoneally with LPS (0.5 mg/kg), and at the given time points, tissues were removed and total RNA was analyzed by Northern blotting (Fig. 2). The expression of xCT mRNA in thymus gradually increased and was highest at 8 and 20 hours after LPS injection. By contrast, in spleen the maximum expression of xCT mRNA was reached already 3 hours after LPS administration. Next, a dose dependency on the expression of xCT mRNA was examined. As shown in Fig. 3, the induction of xCT mRNA in thymus was enhanced with increasing doses of LPS It is noteworthy that the ratio of smaller transcripts (2.5 and 3.5 kbp) to total transcripts was substantially increased in thymus by injection of a dose of 5 mg/kg, which is approximately one fourth of LD_50 _of this kind of LPS. Similar results were obtained in spleen, although the extent of the induction by LPS was much weaker. While the expression of 4F2hc mRNA was scarcely affected by LPS administration in thymus, it was significantly enhanced by LPS in spleen.

**Figure 4:**
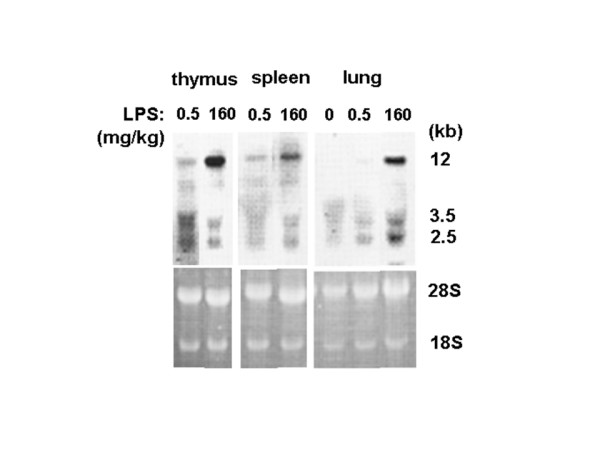
**Expression of xCT mRNA in thymus, spleen and lung of the mice injected with lethal dose of LPS by Northern blot analysis**. Mice were intraperitoneally injected with saline, 0.5, or 160 mg/kg LPS, and the tissues were isolated 5 h after administration. Total RNA was extracted and Northern blot analysis was performed using DIG-labeled RNA probes.

We investigated the effect of the lethal dose of LPS (160 mg/kg) on the expression of xCT in tissues (Fig. 4). In thymus and spleen, strong hybridization signals of xCT mRNA were detected by injection of the lethal dose of LPS. Besides these tissues, a strong signal of xCT mRNA was also detected in lung under these conditions.

**Figure 5:**
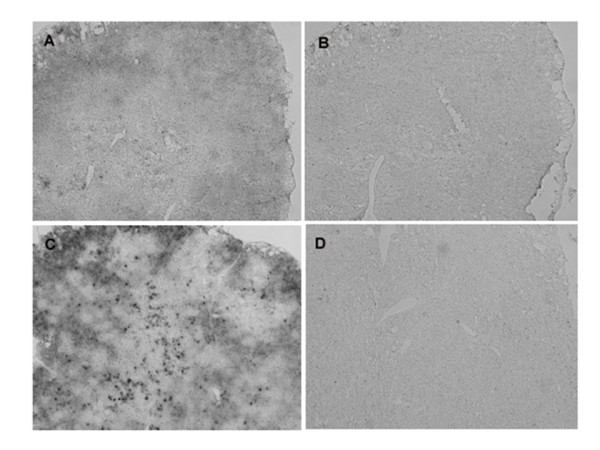
**Expression of xCT mRNA in thymus of the mice injected with a lethal dose of LPS by nonisotopic *in situ *hybridization**. Mice were intraperitoneally injected with saline (A, B) or 160 mg/kg (C, D), and the thymus was isolated after 8 h. Adjacent sections were hybridized with DIG-labeled antisense (A, C) or sense (B, D) probes for xCT. Magnifications: ×50.

**Figure 6:**
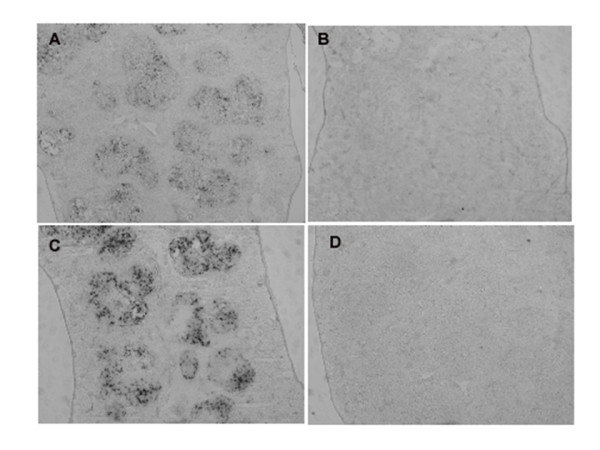
**Expression of xCT mRNA in spleen of the mice injected with a lethal dose of LPS by nonisotopic *in situ *hybridization**. Mice were intraperitoneally injected with saline (A, B) or 160 mg/kg (C, D), and the spleen was isolated after 8 h. Adjacent sections were hybridized with DIG-labeled antisense (A, C) or sense (B, D) probes for xCT. Magnifications: ×50.

**Figure 7:**
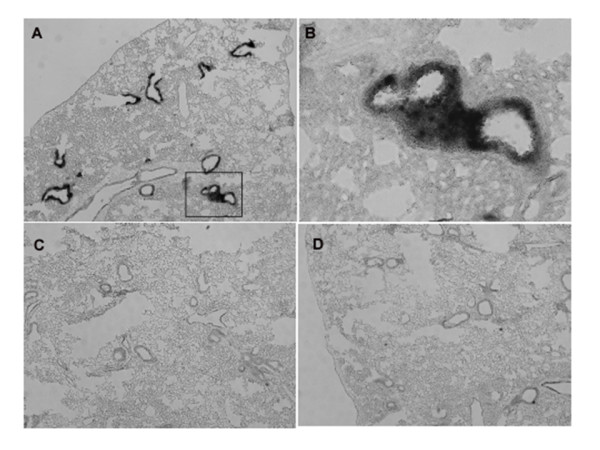
**Expression of xCT mRNA in lung of the mice injected with a lethal dose of LPS by nonisotopic *in situ *hybridization**. Mice were intraperitoneally injected with saline (C) or 160 mg/kg (A, B, D), and lung tissue was isolated after 8 h. Adjacent sections were hybridized with DIG-labeled antisense (A-C) or sense (D) probes for xCT. B is a magnification of the boxed region in A. Magnifications: ×50 (A, C, D); ×200 (B).

Since specific antibodies to xCT for immunohistochemistry have not been available yet, the expression of xCT in the tissues was detected by *in situ *hybridization analysis. The hybridization signals of xCT mRNA were very faint in the thymus and spleen of mice injected even with the sublethal dose of LPS (data not shown). Thus, we performed *in situ *hybridization analyses in the tissues from the mice injected the lethal dose of LPS (160 mg/kg). Under these conditions, the signal of xCT mRNA was greatly augmented in thymus (Fig. 5) and particularly strong signals for xCT mRNA were detected in the cortex of this tissue. Similarly, the signal of xCT mRNA was strongly increased in the white pulp of spleen (Fig. 6). In addition, strong signals of xCT mRNA were detected exclusively in the bronchiolar epithelium of lung in the mice injected with the lethal dose of LPS (Fig. 7).

## Discussion

In the present study, we have detected constitutive expression of xCT mRNA in thymus and spleen in addition to brain [11], and found that LPS is a potent inducer of xCT gene expression in vivo. After administration of sublethal doses of LPS, expression of xCT mRNA was significantly increased in thymus and spleen in a dose-dependent manner. The expression of xCT mRNA was further enhanced by a lethal dose of LPS, and *in situ *hybridization revealed that this expression was mainly confined to the cortex of thymus and to the white pulp of spleen. Under these conditions, we also found that xCT mRNA is massively induced in the bronchial epithelium of the lung.

Mammalian cultured cells expressing xCT transport extracellular cystine via system x_c_^-^, and reduce it to cysteine, which is in turn used for the synthesis of GSH and proteins. A fraction of cysteine is released from cells via neutral amino acid transporters, and the cysteine is rapidly oxidized to cystine. Thus, a series of these transporters and redox reactions constitutes the cystine/cysteine cycle across the plasma membrane [5]. We have demonstrated previously that spleen lymphocytes have hardly detectable activity of system x_c_^- ^regardless of the activation by LPS in vitro [17]. The supply of cysteine for GSH synthesis in lymphocytes may depend on the vicinal cells expressing the activity of system x_c_^- ^and releasing cysteine via the cystine/cysteine cycle into these tissues. Malmezat et al. [18] have reported that the requirement for cysteine is increased in some tissues including spleen during the acute phase of sepsis in rats. GSH synthesis rates are significantly increased in spleen and thymus during the acute phase of sepsis in rats [19] or by LPS administration [20]. The increased GSH synthesis accounts for the enhanced utilization of cysteine at least in part. Thus, the induction of xCT by LPS in spleen may contribute to the supply of cysteine to the lymphocytes by the vicinal cells, although we cannot rule out the possibility that the activity of system xc- is induced in the lymphocytes in vivo.

The activity of system x_c_^- ^sustains the cystine/cysteine cycle, and thus maintains the redox balance between cystine and cysteine in cultured cells [5]. In sepsis patients, the levels of most amino acids in plasma were found to be decreased by 10–30%, whereas cystine and phenylalanine were significantly elevated [21]. Administration of LPS is decreased plasma GSH level [20,22]. We have shown recently that xCT-deficient mice display a significant increase in plasma concentration of cystine and decrease in plasma concentration of GSH, resulting in the oxidative shift towards cystine in the plasma cystine/cysteine ratio [23]. These observations suggest that the organs like thymus and spleen, where xCT is constitutively expressed, contribute to the clearance of cystine in plasma. As a result, xCT may contribute to ameliorating the oxidative shift caused by LPS and maintaining the plasma redox balance. Recent studies have demonstrated that the cystine/cysteine redox balance is associated with aging and proatherogenic events [24,25]. The induction of xCT in vivo may contribute to restoring the plasma redox balance under septic conditions.

The role of glutamate receptors in synaptic transmission and excitotoxicity have been mainly studied in the central nervous system. However, recent evidence points to similar glutamate receptors function also in various other organs, including thymus [26]. System x_c_^- ^is an exchange agency, and the anionic form of cystine is transported in exchange for glutamate with a molar ratio of 1:1 [4]. Thus, the induction of xCT by LPS causes the release of glutamate when cystine is taken up via system x_c_^- ^in thymus. Recently, Pacheco, et al., have demonstrated that glutamate released via system x_c_^- ^expressed in dendritic cells is a highly effective regulator in the initiation of T cell-mediated immune response [16]. Metabotropic glutamate receptors are expressed in mouse thymocytes and thymic stromal cells [27]. Although we have not identified which types of cells express xCT mRNA in thymus in the present study, the glutamate released via system x_c_^- ^into the microenvironment of thymus by the cells expressing xCT might function to activate the glutamate receptors of the cells during T cell maturation and/or proliferation in thymus. The significance of glutamate released via system x_c_^- ^in the immune tissues should be further explored.

Endotoxin induces a whole-body inflammatory response that in turn mediates organ damage. The lung is known to be one of the target organs in which failure is usually apparent. Intense cellular infiltration (predominantly neutrophils) of the interstitium and bronchiolar walls by LPS administration has been reported [28,29]. We have recently demonstrated that the peritoneal exudate neutrophils express xCT mRNA and harbour system x_c_^- ^activity [30]. It is likely that not only the bronchiolar epithelium but also flammatory cells such as neutrophils infiltrated into the bronchiolar walls by LPS administration express xCT mRNA in these areas (Fig. 7). Thimmulappa et al. [31] showed that disruption of Nrf2 dramatically increased the mortality of mice in response to LPS and that the administration of LPS resulted in greater lung inflammation in Nrf2-deficient mice. Their data suggest that Nrf2 regulates the innate immune response during sepsis and improves survival by maintaining redox homeostasis by regulating GSH levels and other antioxidant enzymes through Nrf2. The induction of xCT gene by the stimuli like electrophilic agents is regulated by Nrf2 [10]. However, it has also been demonstrated that induction of xCT by LPS is observed even in Nrf2-deficient mice [32], indicating that the expression of xCT may be mediated through not only Nrf2-dependent but also Nrf2-independent signaling pathways. The activity of system x_c_^- ^is significantly induced by oxygen in human fibroblasts derived from fetal lung [5]. Induction of xCT in lung may be partially responsible for alleviating LPS-induced inflammation by maintaining GSH level.

The results of the present study suggest that the induction of xCT in vivo under the inflammatory conditions may be important to maintain the redox balance in plasma and to supply cysteine for GSH synthesis via the cystine/cysteine cycle to cells, such as lymphocytes. Very recently, Kaleeba and Berger [33] have reported that xCT serves as a fusion-entry receptor for Kaposi's sarcoma-associated herpesvirus. The susceptibility towards infection by this virus may be increased in inflamed tissues where xCT is induced. We expect to gain more insights into the physiological role of xCT under septic conditions in vivo by using xCT null mice.

## Conclusion

The administration of LPS strongly up-regulates expression of xCT mRNA in the tissues like thymus, spleen, and lung. The increased expression of xCT in response to LPS may imply a specific requirement for these tissues to resist oxidative stress conditions caused by LPS and may contribute to ameliorating organ damages in endotoxemia.

## Competing interests

The author(s) declare that they have no competing interests.

## Authors' contributions

KT and MT carried out all the experiments equally. SB and HS supervised the study, participated in its design and coordination, and drafted the manuscript. All authors read and approved the final manuscript.
